# Book Reviews

**Published:** 1876-04

**Authors:** 


### Book Reviews.

[NOTE.—All works reviewed in the pages of the CHICAGO MEDICAL JOURNAL AND EXAMINER may be found in the extensive stock of W. B. KEEN, COOKE & Co., whose catalogue of Medical Books will be sent to any address upon request.]

PHTHISIS. ITS MORBID ANATOMY, ETIOLOGY, SYMPTOMATIC EVENTS AND COMPLICATIONS, FATALITY AND PROGNOSIS, TREATMENT AND PHYSICAL DIAGNOSIS. In a series of Clinical Studies, by *Austin Flint, M.D.*, Professor of the Principles and Practice of Medicine in the Bellevue Hospital Medical College, Fellow of the New York Academy of Medicine, etc. Philadelphia: Henry C. Lea. 1875.

This is a book of 440 pages, divided into six chapters. The first five chapters are devoted mainly to clinical reports of cases of phthisis, 670 in number, which came under the author's observation during a period of thirty-four years. These are grouped and analyzed with reference to their morbid anatomy, etiology, symptomatic events and complications, fatality, prognosis and treatment.

Necessarily, many of the records are deficient in one or more of these elements, so that the various groups are much smaller than the whole number of cases studied.

Thus, in only eighty cases were the post-mortem appearances noted, and in the majority of these only the thoracic organs were examined.

So with regard to the symptomatic events, fatality, etc., many reports are more or less imperfect.

The latter part of the fifth chapter is made up principally of a repetition of the remarks on treatment which are scattered through the preceding pages in connection with analyses regarding various remedial measures. The last chapter treats of the physical signs and diagnosis of the different stages of phthisis.

The purpose of this book as stated in the introduction, is to give the results of clinical studies relating to phthisis.

These results in so far as they are of special interest, as differing from generally received opinions, or in suggesting new thoughts, we epitomize below.

Pulmonary phthisis is a bilateral affection, although it does not fully exemplify the law of symmetry; for both lungs are seldom affected equally, evidently owing to the affection being of older date in one than in the other. The common belief that the left lung is usually first, and therefore most deeply implicated seems erroneous, as shown by fifty-five cases in which the post-mortem condition of the respective lungs was noted. In 28 of these, the greater amount of disease was in the right lung, and in 27, in the left.

Acute miliary tuberculosis usually affects both lungs to about the same extent.

In regard to the relation which granulations, or miliary tubercles, bear to the more common form of phthisis, or tuberculous infiltration, we quote: "It is a reasonable supposition that granulations precede and determine the occurrence of the exudation (which doubtless consists of inflammatory products) within the air cells, the ulterior results being softening, liquefaction and cavities." In this opinion the author differs from those who, following Virchow, believe with Niemeyer, that "The greatest danger for the majority of consumptives is, *that they are apt to become tuberculous*," that is, affected with miliary tubercles. Professor Flint goes on to say: The greatest danger to the majority of consumptives relates to the amount of injury of the pulmonary structure, from exudation, liquefied morbid products, cavities, etc. In a small proportion of cases co-existing miliary tubercles are so abundant that acute tuberculosis seems to have been superadded to the chronic disease. But it does not follow that there is any causative connection between the two affections.

Lobar emphysema is believed to exert a protective influence against phthisis.

Pleuritic adhesions are almost universally found at the autopsy in cases of phthisis, indicating that pleurisy generally accompanies the disease ; and fibroid solidification or interstitial pneumonia occurs in a large per cent. of cases.

A marked tendency to the development of phthisis during a particular decade of life is taken as evidence of the existence of a tuberculous diathesis.

Of 669 cases in which the sex was noted, 505 were males, and 164 females. These statistics differ widely from those of M. Louis, who, in 123 cases observed during a period of three years in a service at La Charité, Paris, found 57 males and 66 females. This variation, as the author remarks, illustrates the liability of statistics to be affected at different times and places by undeterminable extrinsic causes. It farther goes to show the unreliability of conclusions drawn from the analysis of a limited number of cases.

Unfortunately many of the conclusions in this work are based upon the analysis of so few cases as to be of little value ; however, the analyses are interesting and will have increased importance when added to others of a similar character.

We notice what seems to us an error in the author's conclusions, regarding the influence of occupation in favoring the development of phthisis. The occupation was noted in 212 of his private cases. Of these, 30 were physicians ; 32, clerks ; 4, bookkeepers ; 15, merchants ; 14, lawyers ; 11, farmers ; 8, medical students ; 6, clergymen; 7, southern planters; and 5, teachers. In all, seventy different occupations were noted, 46 of which were represented each by a single case ; 7, each by 2 cases ; 2, by 3 cases ; 2, by 4 cases, and the others as shown above.

The author thinks the number of physicians and medical students large from personal reasons. The number of clerks he takes as evidence of an unfavorable influence exerted by their occupation.

In his collection of private cases there was not a single laborer; only one tailor, and indeed, with few exceptions, not more than one following any of the humbler callings. This would seem to indicate that his private cases were drawn from the better classes of society.

In the Chicago City Directory for 1873 and 1874, out of 1,066 names selected at random, we find 80 clerks and bookkeepers, 62 merchants and 148 laborers, beside many tailors, blacksmiths, foundry hands, helpers and others. Adding together the number of clerks, bookkeepers and merchants, we have 142, or a little more than 14 per cent., of the whole number, which still leaves an unfavorable showing for these occupations, if compared with his cases in private practice. However, the average of all persons named in the Chicago City Directory would not be a fair standard by which to compare his private cases, as shown by the fact that among the latter there was not one laborer, while in the directory list there are 148 in every 1,066 whose occupations are given, or about 14 per cent. If we were to take into consideration the number of horseshoers, helpers and others who would naturally be classed as laborers, the per cent. would be greatly increased and would nearly, if not quite, equal that of the laborers in his hospital cases, about 19 per cent.

It seems from these figures that the average of both his hospital and private cases would fairly compare with the average of the directory list.

The whole number of cases in which he noted the occupations, was 370, of which clerks, bookkeepers and merchants constituted 56, or a trifle over 15 per cent.

In the 1,066 names taken from the directory, clerks, merchants and bookkeepers constituted 142, or a trifle over 14 per cent.

It must be remembered that the author's practice has been mostly in cities, and it is probable that the class of persons with whom he came in contact did not vary greatly from the class named in our directory. We find that a little over 14 per cent. of the latter followed in-door pursuits, that is, they were clerks, merchants or bookkeepers, while only 15 per cent. of his cases followed similar occupations. The difference is so trifling, we are forced to believe, that the author's conclusion regarding the agency of in-door life in the production of phthisis is based on insufficient data. His statement is as follows: "As it seems to me, it may fairly be concluded that these facts go to show an agency in the circumstances belonging to the life of clerks and bookkeepers which conduces to the development of pulmonary tuberculosis. The number of merchants may be considered as having, measurably, the same significance." We have purposely reckoned the merchants with the clerks and bookkeepers, as there is no essential difference in their habits with regard to in-door life.

Recalling the fact that the author's cases were mostly seen in city practice, it is interesting to note that the number of farmers was 14 out of 370 names, while in the whole number of names taken from the directory (1,066, there was not one farmer. Cities are full of merchants) and contain but few farmers, yet the number of the latter in this list of phthisical patients is only three less than the number of merchants; *a priori* this would be taken as evidence that of all occupations, farming predisposes most to the development of phthisis. This again shows how easy it is to err in drawing conclusions from statistics. To be accurate, we must not only know the number of persons diseased, but also the number who are healthy; otherwise we might fairly class nationality as a predisposing cause of disease, if in any collection of cases, the per cent. of Germans, Americans or English happened to be large. Prof. Flint holds to the common belief that out-door life, especially such as farmers experience, is in some degree protective against phthisis.

The analysis, so far as it goes, would support an opposite conclusion. It would not seem strange that persons exposed so much to the vicissitudes of the weather, often living poorly, sleeping in small, unwholesome rooms, and frequently working beyond their strength, should be subject to this affection. Statistics gathered from a thousand country practitioners might show a great prevalence of consumption among farmers, for every physician experienced in country practice can recall many who have suffered from this disease.

The author concludes that occupation has an agency in the etiology of this disease, "in so far as it is sedentary and involves confinement within doors." He infers that pregnancy has considerable influence in the development of phthisis, from the fact, that out of 87 females married and under forty years of age, the disease occurred during or shortly after gestation in 22, yet pregnancy seems not materially to affect its course after phthisis has been established.

Pleurisy, bronchitis and pneumonia are not believed to stand in a causative relation to this disease.

Hæmoptysis, when coming from the bronchial mucous membrane and not dependent upon disease of the heart or injuries to the chest, may be considered a forerunner of phthisis, though the latter may not supervene for months or even years.

Contrary to the opinion of Niemeyer, that hæmoptysis is a cause of phthisis, Flint teaches that it is in some degree preventive. After the development of phthisis, hæmoptysis is thought to be a favorable event as regards the arrest or retardation of the disease.

Under the head of treatment, of profuse hæmoptysis, the author refers to the popular belief in the efficacy of sodium chloride, and he admits there may be some reason for it; however, he recommends, chiefly, opiates to soothe the patient, and the cardiac sedatives; among which he enumerates digitalis, aconite and veratrum viride. Digitalis is generally, and we think justly, believed to have an opposite effect on the heart's action from aconite and veratrum; therefore, in cases where the latter are useful, we should expect harm from the administration of digitalis.

Venesection is not advised; in place of it, the author suggests ligation of the limbs. He says: "The effect on the circulation is very great, and without watching, fatal syncope might be induced."

He has employed hæmostatics, such as tannic acid, gallic acid, acetate of lead, pernitrate or persulphate of iron and ergot in many cases, but he finds it difficult to form any positive opinion as to their value, though he does not deny that they may sometimes aid in arresting bronchial hæmorrhage.

He professes to know little of the topical application of astringents. In one instance he saw persistent slight hæmorrhage arrested by inhalation of the vapor of turpentine. This reminds us of Hunter's expression regarding turpentine—"It is the best, if not the only, true styptic."

Persistent diarrhœa occurring during the course of phthisis, is a symptom of ulceration of the small intestines.

Death may result in these cases from peritonitis, induced by perforation of the intestine, or it may be the immediate consequence of profuse hæmorrhage.

Chronic pharyngitis does not denote the existence of phthisis, or a tendency thereto. Chronic laryngitis, which is a common complication of pulmonary phthisis, occurs more often in males than in females. There is reason for believing that it seldom or never precedes the pulmonary affection, and that when it accompanies the latter it does not militate against the chances of recovery, unless it interferes with deglutition.

This complication so often attends those cases of phthisis which progress slowly or are ultimately arrested, that the author considers it a favorable symptom. Permanent huskiness of the voice, or aphonia, is apt to result from laryngeal phthisis, and, unfortunately, these conditions can not be relieved by treatment.

Pneumonia occurring in connection with phthisis, usually terminates in resolution ; it is merely a coincident disease, having no relation to the phthisical affection.

Usually perforation of the lung in consequence of phthisis, is soon followed by death.

Of 24 cases recorded by the author, 21 were fatal ; and the remaining 3 presented symptoms denoting a like termination, but they passed from observation, and their histories are incomplete.

In 10 cases in which the duration was noted, the time from perforation till death averaged a little over three weeks ; individual cases varying from four days to two months.

Thoracentesis may relieve the pneumo-hydrothorax, resulting from perforation, but it can hardly be considered as more than a palliative measure.

Pulmonary apoplexy, gangrene of the lungs, and pericarditis, are among the extremely rare events which occur in connection with phthisis ; they are doubtless only coincident diseases.

The author believes pulmonary phthisis never results from renal disease.

He thinks the rheumatic diathesis (if it has any influence), is antagonistic to phthisis. This view is favored by an analysis of his cases, which gives only three in which the patients were known to have had rheumatism, though the latter is very common in the decades of life in which persons are most exposed to phthisis.

Perineal fistula was noted in 13 cases, in 8 of which the histories were too imperfect to be of value ; but the 5 remaining seem to have been benefited by it ; yet, as the author remarks, it is needless to say they are too few to serve as the basis of positive conclusions.

Of the 670 cases observed, 44 recovered, so that the signs of phthisis entirely disappeared ; in 31, the disease was arrested, so that the patients enjoyed fair health for years after the attack, without any increase in the pulmonary affection ; and in 10, the disease progressed so slowly that life was prolonged after its inception, from two and a half to forty years.

In most of the cases of recovery, there was evidence of only slight consolidation of the lung, and the question may fairly be raised, whether in some of these, other conditions than tuberculous deposits might not have caused the signs.

It may seem presumptuous for us to doubt the judgment of so experienced a diagnostician as the author, but this question has several times forced itself upon us in the consideration of just this class of cases, and we confess to skepticism regarding the diagnosis in several of these forty-four. The fact that a large number of cases, presenting signs indicative of slight consolidation at the apex of one lung, go on to the certain signs of phthisis, may be taken as evidence that similar cases which do not thus progress, are examples of recovery; yet, to us, the evidence seems incomplete.

The author thinks the probability of arrest or recovery from phthisis, other things being equal, is in proportion to the smallness of the deposit; though some cases terminate favorably after the local lesion has become considerable.

Age, sex, family predisposition, hæmoptysis, chronic laryngitis, pleurisy with effusion, and perineal fistula, have little bearing on the prognosis, though, as already stated, some of these may be taken as favorable symptoms.

Essential elements for a favorable prognosis, are good appetite and digestion, and a determination on the part of the patient to overcome the disease.

The duration of fatal cases is somewhat shorter in females than in males. It is likely to be short in females when developed during pregnancy, and in either sex when it occurs after the person has reached thirty years of age. Occupation, and habits as regards the use of alcohol, do not materially affect the duration.

Persistent fever is always a bad symptom, regardless of the amount of the pulmonary affection.

*Treatment.*—Acute miliary tuberculosis tends intrinsically to a fatal termination, and therefore its treatment embraces only palliative measures. Chronic phthisis, that is, the ordinary form of phthisis, has not in all cases an intrinsic tendency to death; on the contrary, in some instances it has an intrinsic tendency to a favorable termination, as shown by the recovery of many cases in which there was no treatment. This the author states succinctly as follows: "Phthisis sometimes ends in recovery, from an intrinsic tendency, without treatment. Hygienic treatment contributes, probably, in no small measure, to this ending. Cod-liver oil and alcoholics, in certain cases, exert a favorable influence."

The duration of the disease was noted in 112 cases; in which it was found to vary from a few months to forty years, the average being about thirty-three months. There was one case supposed to have existed for forty years; one, for thirty-one; and one, for twenty years.

There were 7 cases lasting from ten to fifteen years, and 5 lasting from five to ten years.

The most interesting analysis in the book, is that of 31 cases, in which the disease was allowed to take its course without any treatment of importance, either hygienic or medicinal. Of these cases 8, or about 26 per cent., recovered; in 6, or about 19 per cent., the disease was arrested; and in about 51 per cent. it ended fatally, after a period of from six months to fourteen years.

This analysis is of much importance as a contribution to the study of the natural history of phthisis, and it should make us more modest in our expressions about curing disease, and more careful i our estimation of the value of remedies employed.

There is no error more general or absurd than that of believing recoveries due entirely to the medicines which the patient happens to be taking at the time. This error is so general that even professional men often honestly believe their medicines to have wrought a cure, which was but the natural termination of disease, or the result of non-medicinal causes.

The analysis of 55 cases treated by hygienic measures (under which term the author embraces tonics, palliatives and change of habits as regards out-of-door life), gives 15, or a fraction over 27 per cent., of recoveries; in 10, or a little less than 18 per cent., the disease was arrested; in 7, or about 13 per cent., it was slowly progressive, and in 23, or about 41 per cent., it ended fatally in a short time.

Compared with the preceding analysis, we find the proportion of recoveries greater in this, by a little less than 2 per cent.

The proportion of deaths, as shown here, is a little less, but if we add the number of slowly progressing to the fatal cases in this list as was done in the former, the proportion of deaths is nearly 55 per cent., instead of 41. In this group, the per cent., of cases in which the disease was arrested, is less than in the group which received no treatment. At the first glance, these statistics would seem to show that hygienic treatment was worse than no treatment at all; yet the author thinks "these figures show some influence of hygienic measures on the termination of phthisis, although much less than was to have been expected."

The author concludes that benefit is derived, in many cases from change of habits, involving giving up in-door and sedentary callings and engaging in out-of-door and active pursuits; and in some cases, from a permanent change of residence, in which instances the improvement is more or less due to accessory circumstances; in others, sea voyages and change of climate are especially beneficial. The benefit in this latter instance, seems due more to incidental circumstances than to any special climatic influence, and, therefore, he deprecates the practice of sending patients far advanced in phthisis, away from the comforts of home, perhaps to die among strangers, uncared for and alone.

Chlorate of potassa, alcoholics *moderately*, pancreatic emulsion, and some other remedies, he has used, but not in a sufficient number of cases to render the analyses of much value; however, these remedies appear to possess no special influence in checking this disease. Cod-liver oil seemed to benefit some cases, and so also did the *free* use of alcoholics. The hypophosphites were tried in 16 cases, two of which recovered.

In the general remarks on treatment, the importance of good alimentation is insisted upon; alcoholics, freely, are recommended, when, upon trial, they are found to improve the patient's general condition. Cod-liver oil is recommended, when it is well borne by the stomach; when it is not, some of the other fats may be substituted, and in some of these cases cream will be specially beneficial. In some cases, the cool sponge-bath, daily, seems to invigorate the patient. If we decide upon change of climate, the patient's feelings should be consulted in making the selection. As a rule, mild climates are best for those who are most comfortable in warm weather, and colder climates for those who prefer winter.

Diarrhœa, resulting from intestinal indigestion, is to be treated by tonics. Night-sweating may be checked by some of the remedies in common use for that purpose, and daily paroxysms of fever may often be relieved by anti-periodic doses of quinine.

The author thinks that the marital relations do not operate unfavorably upon the patient; but that children springing from such a union are likely to inherit the tuberculous diathesis. An analysis of these cases fails to furnish sufficient ground for supposing that phthisis can be communicated by the husband to the wife, or *vice versa*, through the intimate relations of married life.

We, therefore, conclude that the only evil likely to result from the marriage of phthisical persons falls upon their descendants.

The author asks: "Shall the physician approve of the marriage of phthisical patients?"

He gives the conclusions drawn from his analyses, but leaves the reader to answer the question according to his own sense of morality and justice. Individually, we believe it wrong to inflict needless injury upon the living or the unborn, and in view of the facts presented, we hope the profession and the public will answer this question with a decided NO.

The last is a well-written chapter, treating of the physical signs and diagnosis of phthisis. Its teachings do not materially differ from those of other authors, but a few minor points are noticed which we do not remember to have seen in other books. Among these we find the following:

Cracked metal and amphoric resonance may sometimes be elicited over the primary bronchi when the intervening lung is solidified.

Neither whispering pectoriloquy, nor pectoriloquy with the loud voice, are distinctive of cavities, because articulate words may be transmitted by solidified lung. The pectoriloquy produced in cavities may be distinguished from that occasionally obtained over solidified lung by being low-pitched and blowing, while the latter is high-pitched and tubular.

The author describes cavernous, and broncho-cavernous respiration, and cavernous respiration with an amphoric intonation.

The first of these is pathognomonic of a pulmonary cavity. It is a low-pitched non-vesicular puffing or blowing sound, heard during both portions of the respiratory act.

A few typographical errors occur in this as in most other books; thus, in the record of a case (p. 86), it is stated that he had not lost in *height*, and similarly (p. 257): "The heart was enlarged, weighing fourteen pounds."

Though the analyses which are given in this work have been carefully made, we can not but think the number of cases in each too small to justify definite conclusions; yet we feel under obligation to the author for leaving to us the results of his labor and large experience, for we think the histories of these cases will become of great importance when compared with many more of the same character.

The publisher has perpetrated another nuisance by binding with this work an advertising pamphlet, which interests only himself. We think it outrageous that any one who wishes to place this work in his library, must store with it such a quantity of trash.

Handbills are useful in their places, and may possibly be ornamental on fences and neglected walls, but none of us care to cover our libraries with them.

We hope publishers will soon learn to send us their advertisements in a more agreeable manner. E. F. I.

THE BEST WELFARE OF INVALIDS SEEKING THE BENEFIT OF CLIMATE; with Suggestions for the Co-operation of Physicians, Life Insurance Officials, etc. By *Charles Denison, M.D.*, Denver, Colorado.

This brochure of Dr. Denison is full of practical suggestions, and its perusal must lead us to profitable reflections on change of climate. Among other things he says: "The facts and statistics, the results of experience, which are to constitute the storehouse of knowledge in this important direction, are yet to be furnished by faithful observers. The basis of climatic treatment, if there has been any, has hitherto been largely furnished by interested parties whose mercenary motives have greatly affected their influence. The Kansas Pacific Railroad, for instance, with the business zeal characteristic of railroad enterprises, has expended over a hundred thousand dollars in advertising Colorado, chiefly as a health resort. This has been done in a manner not very dissimilar to the way a patent medicine vender would advertise his claims, yet, perhaps, with not as much injury as he would do if working on as large a scale. * * * * * Most desirable statistics, of just how many do annually journey far for health, are wanting. We have only newspaper reports of so many being *here* and so many *there*, a number spending the winter in one locality or the summer in another."

In the absence of the definite knowledge which an accumulation of statistics would afford us, it is not strange that mistakes should be committed in selecting a climate for invalids.

The Doctor points out other errors of great moment in the following paragraphs:

"A point of great importance in this connection is, that of those who do resort to healthful climates for regaining lost vitality, many are constantly perpetrating this mistake, that simply *to be* in the vicinity of the health resort is sufficient, while the temper of the mind, the out-door life, and the blessed sunshine, are disregarded. I can not better express my opinion of the necessity of some supervision over the habits of thought and the separation of consumptives from each other, than to quote from an excellent letter from Dr. D. I. Caine, of Asheville, N. C. He says:

"'I think it is a great and grave error for persons of wealth, intelligence, refinement and high nervous organization to be congregated in buildings at different health resorts, whether they be called hospitals, hotels, boarding houses, etc. The spectacle to each other, of so many afflicted with the same or similar disease; the sight of the dying, reminding them of their own (nearly certain) end; the staple subject of talk being the amount of expectoration, the degree of cough, of sweat, etc., etc.; all these tend to exert a very depressing influence upon all. So far from advising *ag*gregation, I have invariably advised *se*gregation.'

"There is often, too, a lack of confidence and mutual dependence between physicians widely separated, which I can not help thinking sometimes lessens the patient's chances of recovery. Who can be expected to know more of the conditions of life and success in treatment, at a far distant health resort, than the observing physician whose field of work is there? In this connection let me quote from an interesting letter from a faithful observer, Dr. Manning Simons, of South Carolina:

" 'I am perfectly satisfied that a more careful consideration is due to the application of climate to pulmonary disease than the subject receives. Too much is trusted to chance and experiment in individual cases. A physician sends his patient away for a change, not with any definite idea as to the indication to be met by any particular climate, but all of them to the same region, that most fashionable at the time. Most of the people from the Northern and Eastern parts of the country, who make up the largest part of those who travel in the South in search of health, seldom consult Southern physicians, but prefer to communicate, even by letter, with their own medical attendants, who, though at a distance, are presumed to know more of this matter than those of us who have looked especially into the subject.' "

As a means of procuring extensive statistical information, and properly making use of it for the benefit of the invalid, the Doctor proposes a Climatic Association, composed of physicians interested in such labor, and especially representatives from the health resorts of America, devoted to the prolongation of life and the adaptation of climate to the needs of invalids. It should be their work to gather statistics of all climates, familiarize themselves with the details of their special labors, and to tabulate all the results of the journeyings of invalids in America from now henceforth.

This association should always seek to prevent the *useless* and encourage the *useful* migrations of invalids, and continually watch over the journeyings of their patrons.

Insurance companies could, and the Doctor thinks would, avail themselves of the knowledge gained through this association, to send their invalid policy holders to suitable climates for the prolongation of their lives.

Through a central council or bureau of medical advisers, communication with the rest of the medical profession, insurance companies, and invalids generally, could chiefly be carried on by means of a specially prepared medical examination or diagnosis paper, and in return advice could be given as to the choice of climate, mode of life needed, etc., etc.

Such, in brief, is the plan proposed for consideration. Let us, by way of illustrating how this scheme would work, suppose a single case.

A. B., of Maine, for instance, is in failing health, his case is complicated and he has reason to fear consumption. Either from a previous knowledge of the Climatic Association, or by the counsel of his physician, he writes to the Advisory Bureau. The examination papers are sent to A. B. (or his physician) with advice, if needed, as to what physician should make a medical examination of his case. The examination papers are filled out, partly by the physician and partly by A. B. himself. These are returned to the Advisory Bureau. These are full and explicit, giving particulars upon all points which should be inquired into. Thus, with the medical facts, personal history, desires, pecuniary circumstances, etc., of the applicant before them, the head-physician can advise the mode of life and change of climate, etc., needed, better than any physician alone, whose time is taken up with busy general practice.

Transactions of the Colorado Territorial Medical Society, at its Third and Fourth Annual Sessions, held in Denver' June, 1874, and June, 1875.

The transactions for 1874 are very briefly reported in the volume before us, most of the papers having been published elsewhere. In 1875 the report is more full. There is a very interesting report on Gynecology, by Dr. Newman, wherein twelve operations by different surgeons are recorded ; a paper on Blood-letting, by Dr. McClelland, in which strong ground is taken in favor of the more frequent employment of this medical resort ; a report on Climatology, by Dr. T. E. Massey, of Denver, which is interesting and novel, as coming from a Colorado doctor, by his declaration that, while in the climate of the Territory consumptives and asthmatics get a new lease of life, and sufferers from malaria are sick with this annoying malady nevermore—that, "the wrinkled skin of middle-aged men, and the tallow faces of youngish maids and matrons are most significant, that the *drying process* of this elevated region is promotive of neither longevity nor beauty."

Here is a report of two cases of rheumatism—one acute, the other sub-acute—successfully treated with tight bandaging over a thick layer of cotton wool, by Dr. Gehrung; a paper on The Early Symptoms and Signs of Phthisis, by Dr. Lemon; a brief paper on Congenital Cataract, by Dr. Chas. Denison; while Dr. F. J. Bancroft reports a case of vomiting of pregnancy, which became so severe and continued so long—to the end of the third year—that the patient became typhoid in her debility and depression. All efforts for relief proving useless, the first step toward the production of miscarriage—the introduction of a sponge tent into the neck of the uterus—was resorted to. As soon as the uterine neck began to be expanded the sickness ceased, the sponge was soon after withdrawn, and the patient completed her gestation without nausea or other inconvenience, and was delivered of a healthy child.

The volume closes with reports of cases—one of paraphymosis, by Dr. H. A. Lemon and by Dr. W. H. Williams, one of tumor in the hypogastrium, and one of strangulated umbilical hernia.

Altogether, the society may fairly congratulate itself on its growth, and the fact that it is doing substantial work.

The society has recently been incorporated.

A Practical Treatise on Fractures and Dislocations. By *Frank Hamilton*, *A.M.*, *M.D.*, *LL.D.*, Surgeon to Bellevue Hospital, New York; Consulting Surgeon to Hospital for Ruptured and Cripples; to St. Elizabeth Hospital, etc.; author of a Treatise on Military Surgery and Hygiene, and of a Treatise on the Principles and Practice of Surgery. Fifth Edition. Revised and Improved.

This treatise is so well known to the profession that its fifth edition certainly requires but a short notice at our hands. It would be idle, at this time, to commend a work, which, for years, both at home and abroad, has been looked upon as the highest authority. The number of pages and wood-cut illustrations have been increased. In scanning the pages of the new edition, we notice (page 117), an example of *permanent* paralysis of the inferior dental nerve, from fracture near the angle of the bone. This case is an exceptional one, the nerve being seldom injured. In the other cases reported, the paralysis was of short duration.

In referring (page 205), to the difficulties of maintaining the two fragments of a broken clavicle in the original line of axis, the author objects to the opinion held by some surgeons, that it is quite as important to depress the sternal fragment, as it is to elevate the acromial. The accomplishment of this object is attempted, (1), by the inclination of the head toward the injured side ; (2), by compresses and adhesive straps. The patient tires of the former, and the latter is inefficient. To Dr. Moore's method (bringing into requisition the clavicular fibres of the pectoralis-major, with the view of antagonizing the action of the sterno-cleido-mastoid, by thrusting the elbow behind the body), his objections are both practical and theoretical.

The practical objection is, that the dressings required to maintain the arm in position are exceedingly liable to produce excoriations. The results are no better than are realized from the use of his (Hamilton's) own dressings.

The theoretical objection is, that the clavicular fibres of the sterno-cleido-mastoid will soon become relaxed under the continual strain, and after a time, cease to accomplish what they did at first. Prof. Hamilton also suggests that if the pectoral muscle is thus rendered less competent to depress the sternal end of the bone, the sterno-cleido-mastoid will be rendered likewise less competent to elevate it, making some allowance, as he does, for the different angles at which these two muscles act upon the bone.

Moore's method, in our opinion, can not effect a "continual strain upon the sterno-cleido-mastoid muscle;" but whilst it does effect this in the case of the clavicular fibres of the pectoralis-major muscle and thereby produce more or less traction on the sternal fragment of the fractured clavicle, it has no power in the case of the former muscle to enforce the well-known law in regard to the action of muscles put upon the strain. The author declares his preference for an apparatus consisting essentially of a sling, axillary-pad, and bandages to secure the arm to the chest, the arm hanging perpendicularly beside the body.

If we add to these the "posture treatment," when the danger of disfigurement would warrant a resort to it, the several indications seem to be fairly met. Fortunately, overlapping and deformity do not cause diminution of the strength or usefulness of the arm, except *in those cases where the cupidity* of the patient has been excited, and then "*the weakness*," "*the coldness*" and "numbness" of the arm can be cured by one specific only—money!

In the chapter on fractures of the scapula (page 214), Prof. Hamilton employs the terms "anatomical" neck, and "surgical" neck, the former being applied to that slightly constructed portion situated at the base of the glenoid cavity, and the latter to the part of the bone corresponding to the root of the coracoid process at the site of the semi-lunar notch.

The treatment of fractures of the surgical neck of the humerus, including separations at the epiphysis (page 242), is given at greater length. The author now recommends an additional splint to extend from the free margin of the axilla to the internal condyle. Its purpose is not to support the fragments, since it can not extend so high, but to protect the skin from injury by the bandage. He considers as wholly unnecessary, the bandage applied by some surgeons to the hand and forearm.

Prof. Hamilton has been somewhat minute in his description of this dressing, since its value, he claims, depends upon the care with which the details are carried out.

We think that the author has, in this fifth edition, done the profession a service by indicating the methods by which a faithful measurement of the arm or forearm (page 256), and thigh (page 418), may be made. He recognizes not only the weak point in Bryant's suggestions to measure by the ilio-femoral triangle, in order to determine a fracture of the neck of the femur ; but also the unsoundness of Velpeau's idea to measure from the folds of the belly. Doubtless the author has had abundant opportunities of noting the ease with which inaccurate measurements may be made.

In the chapter on fractures of the radius, there is some additional evidence that we have not yet attained to all that is desirable in the treatment of Colles' fracture. Moore's method has given no better average results than have been obtained by other modes of management.

The pages devoted to the treatment of fractures of the femur, tend to settle us more firmly in our convictions that plaster of Paris, as an immovable form of dressing, is by no means as safe as Buck's or Hamilton's apparatus. As ordinarily used, plaster of Paris is safe only in the hands of the experienced surgeon, it being highly injudicious to advocate its general adoption. Employed after the "book-back" or Bavarian method, it becomes more manageable. Upon the authority of Prof. Hamilton, the plaster is thus used by Dr. Paul F. Eve, Professor of Surgery, Nashville Medical College. Extension and counter-extension are made as in Buck's method, and the limb is exposed to view daily and sponged.

Part Second contains some new matter. Dr. John T. Darby, (page 586), by adopting a rule similar to that which the author has laid down, in reducing dislocations of the thigh, namely, by carrying the arm only in those directions in which it meets with the least resistance, has been very successful in reducing dislocations of the shoulder.

At page 600, the new edition gives the following summary of accidents growing out of efforts to reduce old dislocations of the shoulder :

"Rupture of an artery, nineteen cases, most of which were known to be ruptures of the axillary artery. Callender, Lister and Blackman tied the axillary, and the patients all died. The subclavian was tied by Warren successfully. Gibson also tied the subclavian, but his patient died. Nélaton did the same—result not stated."

"Rupture of vein alone, two cases. Froriep's patient died; Agnew's was saved."

"Rupture of artery and vein, probably two cases. Platner's patient died. In Bell's case the result is not stated, except that amputation was practiced."

"Avulsion of arm, one case. Patient died."

Of fractures of the neck of humerus, the author has reported three cases. In none of them was reduction accomplished. His own patient died.

The difficulties which so frequently beset us in our efforts to reduce a certain proportion of these old dislocations of the shoulder, the author attributes to the changes which may take place in the socket and in the adjacent tissues. That a diminution in the size of the rent in the capsular membrane may resist the reduction of the bone, we saw illustrated not long since, at the Rush College Clinic, Chicago. A man with a dislocation of the shoulder of six weeks' standing, presented himself at the college clinic. Prof. Gunn, after failing in every effort at reduction, including extreme circumduction for the purpose of lacerating the ligament and breaking up adhesions, cut down through the deltoid into the joint, and found the rent in the capsular so much contracted as not to admit the return of the bone to the socket. After sufficiently enlarging the rent with a probe-pointed bistourie, the reduction of the bone was easily effected.

J. E. O.

Syphilitic Lesions of the Osseous System in Infants and Young Children. By *R. W. Taylor*, *M.D.*, Physician to the Charity Hospital, N. Y., etc. New York: Wm. Wood & Co., Publishers. 1875.

He is a zealous adventurer who, in the present day, makes a voyage of discovery, where every nook and corner has been repeatedly explored before. Such, however, has been the task of the author of this treatise. Long familiar with all the recognized varieties of venereal disease, he has discovered in the osseous system of infants a grave and distinct form of lesion, heretofore overlooked, or recognized only in the post-mortem condition.

Dr. Taylor observed his first perplexing case in 1867; and could then find in medical literature but a few details, given by Rannier, descriptive of its pathology. In the ensuing year Wegner published a description of the microscopic appearance of the bones of a number of syphilitic children, exhibiting peculiar pathological phenomena; and, within the two following years, Waldeyer, Köbner and Parrot confirmed his observations, without, however, contributing to the clinical history and therapeutics of this form of disease.

The basis of the present work, which is really a continuation of the general subject, is a collection of clinical cases, valuable in themselves, and made of greater interest in consequence of the faithfulness evinced in their description. This collection includes not only the cases observed by Dr. Taylor, but those also of the observers referred to, and others.

Beginning methodically with the radius and ulna, the general characters of the changes in these bones are found to be those which are presented when the disease is elsewhere developed. If the outlines of these parallel shafts be traced downwards with the tips of the fingers, an abrupt circular elevation is discovered surrounding each, except at the point of osseous contact. This may be smooth or undulating, and limited to the shaft, or encroaching upon the epiphysis to about two-thirds of its extent; when there is complete fusion of the epiphyses, it is the result of excessive cell-proliferation in the fissure between them. At the upper extremities of these bones this condition is less distinct, in consequence of the disposition of the parts, except at the olecranon. One or both bones may be affected, and may or may not present the swelling in common with the lower ends, in comparison with which they are less liable to become involved.

The humerus in general escapes. Dr. Taylor has obseved the only case hitherto described in which the sternal end of the clavicle has been attacked. In rare instances a bulb-like projection, with an even outline, is encountered on the surface of one or more ribs.

Abrupt swellings, elevated to the extent of one-half or three-fourths of an inch, involving the shaft or merging into the epiphysis, are encountered about two inches above the malleoli. If circular, the bones are completely surrounded, their extremities remaining intact, but the resulting deformity being greater than at the wrist. Tumors of this kind at the ankle-joint are frequent, involving one or both legs, and attacking both bones or limited indifferently to either. These lesions have, in each instance, co-existed with others of the same class, especially in the fore-arm. Of the fourteen cases observed by Taylor, but one occurred with involvement of the superior termination of the tibia, and one also of the trochanter of the femur, the soft tissues surrounding this bone presenting an obstacle to its proper exploration. All the distal phalanges have been observed affected in this manner, the fingers presenting a bulbous and clubbed appearance, suggestive of syphilitic onychia. The central phalanges seem to possess some immunity, as no instance of their involvement has been described, though the author has observed an hyperplasia in this locality which might serve as a type of the disease, when thus developed. The proximal are more liable to be attacked, in the proportion of five to one, than the distal phalanges, the deformity being acorn-shaped and two or three times greater in size than the normal osseous tissue. The integument thus stretched may ulcerate, and this accident may be due to degeneration in the thickened bone itself. No instance has occurred of this disease in the phalanges of the foot, though there is no reason to conclude that they possess any special immunity. The fourth and fifth metatarsal bones have been observed affected in a similar manner, with this peculiarity, that the entire length of the bone has been involved, with greatest development in the middle of the shaft. Of the flat bones, lesions of the cranial are infrequent—in four instances the parietal, and in three the occipital, have been affected; a behavior in striking contrast to that of hereditary syphilis in later life.

The clinical history, development, course, decline and concomitant symptoms of the disease are carefully sketched. In one class of cases there is rapid development of the lesion, in another it occurs with comparative slowness—the average period being from two to six weeks; the shortest, two weeks; the longest, two months. Uncomplicated cases, properly treated, rapidly assume the phases of repair. Degeneration may be superficial or deep—rarely the epiphysis becomes separated from the diaphysis. When a sinus forms, it gives exit to a secretion of a light brown color, which may be followed by rapid restoration of the normal conditions. Inasmuch as the tissues are yet yielding, and the disease is one of defective nutrition, pain is not severe. The constitutional disorder, in fact, is one which is early engrafted upon the growth of the fœtus in the period of active development of all its tissues. Deep disorganization has been observed only in the still-born, the survivors may not present symptoms of the vice before the third week, and possibly not before the second or third year.

The author is at issue with Waldeyer and Köbner respecting the possibility of the development of these accidents in the acquired syphilis of infants. One such case Taylor has described, and, while admitting that this late occurrence may be less destructive to the tissues, he concludes that there is nothing in the immaturity and rapid evolution of the fœtal tissues which renders them exclusively susceptible to the morbific agency.

Three pathological stages are distinct: 1st, Exuberant cell-proliferation; 2d, Irregular osteogenesis; 3d, Abnormal proliferation, with inter-cellular infiltration.

Cachexia, from whatever cause, predisposes to rickets, and one of these causes is undoubtedly the syphilitic diathesis, but the symptoms and treatment of rickets, which is of later appearance than hereditary syphilis, are not similar.

Medical works too often conclude with an anti-climax, which results from the unequal advance of pathology and therapeutics, but the volume before us is a worthy exception to the rule. Our author employs mercury with the potassic iodide, continuing the medication for a long period if no improvement results. Salivation is never induced, nor a mercurial cachexia, the health being actually improved by pursuing this course, without having recourse to tonics, which, however, are not to be neglected. Lewin's method of hypodermic medication as well as inunction are discountenanced. Ulcers are sprinkled with iodoform.

The author concludes with a consideration of the tumors occurring later in life at the junction of the epiphyses with the diaphyses, as well as the more specific affections of the osseous system, simulating syphilis.

We regard the work, as a whole, in the light of an exceedingly valuable contribution, not only to the special department to which it might be assigned, but also to the general literature of medicine. We congratulate the author upon the evidences it contains of his scholarly and conscientious labor, which have already secured for it the encomiums of its most judicious critics.

### BOOKS RECEIVED.

A Manual of General Pathology. By *Ernst Wagner*, *M.D.*, Prof., etc., University of Leipzig.

Medical Diagnosis, etc. By *J. M. Da Costa*, *M.D.*, *etc.* Fourth edition, revised.

Inhalation in the Treatment of Disease, etc. By *J. S. Cohen*, *M.D.*, *etc.* Second edition, revised, etc.

Medical Thermometry and Human Temperature. By *E. Seguin*, *M.D.*

Transactions of the Pathological Society of Philadelphia. Volume fifth. Edited by *Jas. Tyson*, *M.D.*, *etc.*

Transactions Illinois State Medical Society for 1875.

A System of Midwifery, etc. By *Wm. Leishman*, *M.D.*, *etc.* Revised edition.

A Treatise on the Diseases of Infancy and Childhood. By *J. Lewis Smith*, *M.D.*, *etc.* Third edition, revised.

HOSPITAL PLANS: Five Essays on Hospital Construction, etc. JOHNS HOPKINS HOSPITAL.

HUMAN PHYSIOLOGY, ETC. By *W. B. Carpenter, M.D., etc.* New and revised edition.

INSANITY IN ITS MEDICO-LEGAL RELATIONS. By *A. C. Cowperthwait, M.D.*, Kansas City.

THE MEDICAL JURISPRUDENCE OF INSANITY. By *J. H. Balfour Brown, Esq.*

SURGERY: ITS PRINCIPLES AND PRACTICE. By *T. Holmes, M.A., Cantab., etc.*,

EXTRA-UTERINE PREGNANCY, ETC. By *J. S. Parry, M.D., etc.*

PAMPHLETS RECEIVED.

THE SANITARY CONDITION OF BOSTON—Report of a Medical Commission, etc., 1875.

TRANSACTIONS OF THE NEW JERSEY MEDICAL SOCIETY, 1766 to 1800.

ON ALCOHOL: A Course of Lectures, etc. By *B. W. Richardson, M.A., M.D., etc.*

PROCEEDINGS OF "AMERICAN ASSOCIATION FOR THE CURE OF INEBRIATES," at Hartford, Conn., Sept., 1875.

THE CAUSE OF THE COMMENCEMENT OF PARTURITION. By *Charles M. Crombie, M.B., M.C.*, of Aberdeen, Eng. From J. Penington & Son, Phila.

THE PRINCIPLES OF PHYSIOLOGICAL ANTAGONISM, AS APPLIED TO THE TREATMENT OF THE FEBRILE STATE. By *Roberts Bartholow, M.D., etc.* This is No. 1 of Vol. II of "A Series of American Clinical Lectures."

ON THE ADMINISTRATION OF DIGITALIS IN THE WEAK-HEART OF CONTINUED FEVER. A Paper by *E. T. Easley, M.D., etc.*, Little Rock, Ark.,

ON AUSCULTATION OF THE ŒSOPHAGUS. A Paper by *Louis Elsberg, M.D., etc.*, New York.

APHONIA: ITS CAUSES AND TREATMENT. A Paper by *William Porter, M.D.*, St. Louis.

---

ENCOURAGING!—The January numberof the *Journa de Médecine et de Chirurgie Pratiques*, extracts from the *Philadelphia Medical Times* and the *Practitioner*, Prety's observations on the treatment of sciatica, originally published in the *Wiener Medicinische Presse.*

When the French journals look for the latest scientific researches of the Germans in American medical periodicals, we may well believe that the world moves.

---

DR. STEINER, of Prague, the well-known writer on diseases of children, died February 15.

THE SIXTH ANNUAL COMMENCEMENT EXERCISES of the Woman's Hospital Medical College, of Chicago, were held on February 29th, at the First Methodist Church, on which occasion the following ladies received the diploma of M.D.:

MRS. MARY H. BOWEN, Illinois.
MRS. LOUISA M. GRONARD, Illinois.
MRS. LOIS FITCH MANSFIELD, Illinois.
MISS MARGUERET CALDWELL, Wisconsin.
MISS EVA BICKFORD, New Hampshire.
MISS AMIE M. HALE, Massachusetts.
MISS HARRIET E. GARRISON, Illinois.
MISS AMANDA M. RANSLOW, Wisconsin.
MISS ADELIA BARLOW, Illinois.
MISS HANNAH C. RUSSELL, Wisconsin.

